# External stimulation induces the secretion of autophagosome-like vesicles by B cells

**DOI:** 10.1080/27694127.2023.2179287

**Published:** 2023-02-21

**Authors:** Yu-Diao Kuan, Chao-Yuan Tsai, Shuhei Sakakibara, Daron M. Standley, Hitoshi Kikutani

**Affiliations:** a Department of Genome Informatics, Research Institute for Microbial Diseases, Osaka University, Suita, Osaka, 565-0871, Japan; b Laboratory of Immune Regulation, Immunology Frontier Research Center, Osaka University, Suita, Osaka, 565-0871, Japan

**Keywords:** Autophagosome-like vesicles, B-cell activation, CD40 receptor, Extracellular vesicles, IL-4, RAB27a, Secretory autophagy

## Abstract

Macroautophagy/autophagy is a cellular degradation and recycling process that supports cellular homeostasis. Since an autophagosome marker, microtubule-associated protein 1A/1B-light chain 3 (LC3)-II, was found in cell-derived extracellular vesicles (EVs), autophagy may cooperate with EV secretion pathways to control unconventional secretion of intracellular molecules. Several studies have demonstrated that pharmacological inhibition of autophagic turnover and pathogen-induced endolysosomal dysfunction enhanced the secretion of autophagosome-like EVs (ALVs). However, whether external stimulation induces ALV secretion is unclear. Here we showed that co-stimulation with IL-4 and anti-CD40 antibody (IL-4:CD40) enhanced the secretion of LC3-II^+^ALVs compared to co-stimulation by IL-4 and lipopolysaccharide (IL-4:LPS) or by IL4 and anti-IgM antibody in B cells. While IL-4:LPS stimulation accelerated autophagic flux, IL-4:CD40 stimulation reduced autophagosome-lysosome fusion without affecting lysosomal function. Although both IL-4:LPS and IL-4:CD40 induced the expression of similar genes involved in vesicle fusion or transportation, IL-4:CD40 preferentially enhanced the expression of the small GTPase RAB27a compared to IL-4:LPS. Genetic disruption by the CRISPR-Cas9 system revealed that loss of RAB27a membrane-binding ability impaired LC3-II^+^ALV secretion but not ALIX^+^EV secretion in B-lymphoma A20 cells. Additionally, reconstitution of human wild-type RAB27A in RAB27a mutant A20 cells restored LC3-II^+^ALV secretion, indicating that RAB27a controls autophagosome secretion. Furthermore, LC3-II^+^ALVs were found in the sera of tumor-bearing mice and the plasma of healthy human donors. Our findings may provide a role for B-cell secretory autophagy in regulating intercellular communication under various physiological conditions, such as vaccination, pathogen infection, and B-cell lymphoma progression.

**Abbreviations:** ALVs: autophagosome-like vesicles; ATG: autophagy-related; Baf A1: bafilomycin A1; CNX: calnexin; EVs: extracellular vesicles; Ig: immunoglobulin; IL: Interleukin; LC3: microtubule-associated protein 1A/1B-light; LPS: Lipopolysaccharides; MVs: microvesicles; RAB: member RAS oncogene family; TLR: toll-like receptor

## Introduction

Macroautophagy (hereafter referred to simply as autophagy) is a fundamental intracellular degradation system that contributes to cellular homeostasis and host defense [1-3]. External stimulation, cellular stress, and pathogen invasion can each induce the initiation of phagophore formation [4]. The phagophore elongates and closes to form a double-membrane organelle called an autophagosome, which encloses cytosolic cargo [4]. Subsequently, the outer membrane of the autophagosome fuses with lysosomes to form an autolysosome, which results in the degradation of its contents and inner membrane by lysosomal enzymes [4]. In addition to the autophagic degradation pathway, recent studies showed that suppression of autophagosome-lysosome fusion enhanced the secretion of extracellular vesicles (EVs) carrying autophagy-related (ATG) proteins [5,6] such as LC3-II or SQSTM1/p62 (hereafter referred to as p62). Pathogen infection-induced lysosomal dysfunction also resulted in the secretion of autophagosome-like vesicles (ALVs) [7-9]. These findings indicate an expanded role of secretory autophagy in regulating biological processes such as pathogen exocytosis, intercellular communication, and cellular quality control. However, ALV secretion is likely to occur only when autophagy turnover is inhibited [5-7,10]. Moreover, it is still unclear whether cells exposed to environmental stimuli secrete ALVs.

During infection or antigen immunization, antigen-experienced B cells migrate to the border between the T-cell zone and the B-cell follicle, where they are fully activated by interacting with T cells that express CD40 ligand (CD40L) and other co-stimulatory molecules [11,12]. It has been reported that T cells induce the B-cell secretion of exosomes carrying major histocompatibility complex class II through CD40L-CD40 interaction [13,14]. IL-4, a T helper type 2 (Th2) cytokine, also activates autophagy in B cells [15]. Thus, activated B cells may secrete ALVs during B-T cell interaction. In the present study, we demonstrate that external stimulation induces ALV secretion by B cells. Compared to IL-4 and toll-like receptor (TLR)-4 co-stimulation-induced high autophagic flux, IL-4 and CD40 co-stimulation induces both autophagosome and lysosome formation, but reduces their fusion. The accumulated autophagosomes are secreted through a small GTPase (RAB27a)-dependent exocytotic trafficking pathway. Our findings provide the first evidence that external stimulation induces the secretion of ALVs by B cells. For this reason, B-cell secretory autophagy may play an important role in the regulation of intercellular communication.

## Results

### External stimuli induce the secretion of ALVs by B cells

**Figure 1.**
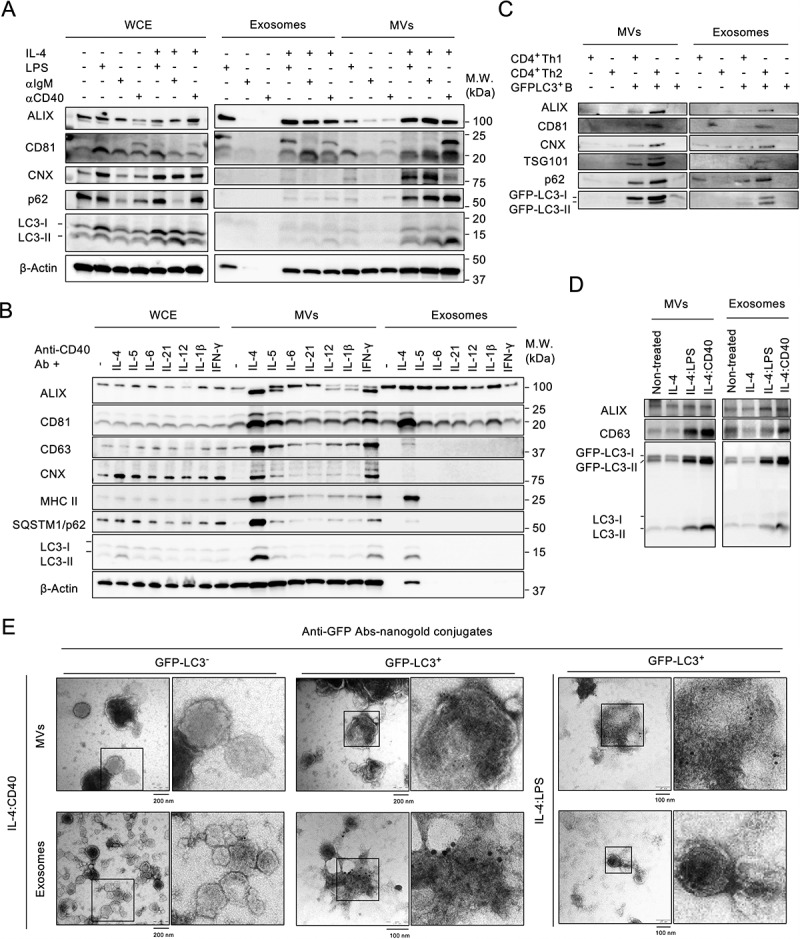
External stimuli induce the secretion of ALVs in B cells **(A)** Equal numbers of primary B cells were stimulated with the indicated stimuli for 4 days prior to isolation of the exosomes and MVs from supernatants. Whole-cell lysate (WCE), isolated exosomes, and MVs were analyzed by immunoblotting using antibodies (Abs) against ALIX, CD81, CNX, p62, LC3, and β-Actin. **(B)** Equal numbers of primary cells were stimulated with various cytokines combined with anti-CD40 Ab for 4 days. WCE, isolated exosomes, and MVs were analyzed by immunoblotting using Abs against ALIX, CD81, CD63, CNX, MHC II, p62, LC3, and β-Actin. **(C)** GFP-LC3^+^ primary B cells were co-cultured with Th1 cells or Th2 cells at a ratio of 1:1. MVs and exosomes from indicated culture condition were analyzed by immunoblotting using Abs against ALIX, CD81, TSG101, CNX, p62, and LC3. **(D)** Equal numbers of GFP-LC3–A20 B cells were stimulated with the indicated stimuli for 24 h prior to the isolation of exosomes and MVs from supernatants. Isolated exosomes and MVs were analyzed by immunoblotting using Abs against ALIX, CD63, and LC3. **(E)** Representative TEM images of MVs and exosomes from GFP-LC3^+^ primary B cells stimulated with IL-4:LPS or IL-4:CD40. EVs were prepared as in **(A)** and **(B)**. GFP-LC3 was probed with anti-GFP Abs and labeled with 10-nm nanogold-conjugated secondary Abs. EVs from GFP-LC3^−^ primary B cells were used as staining control. Scale bars, 100 nm. All experiments were repeated at least twice and showed similar results.

We first investigated whether external stimulation induced ALV secretion by B cells. Splenic naïve B cells were co-stimulated with IL-4 and one of the following: anti-CD40 antibody (IL-4:CD40), lipopolysaccharide (IL-4:LPS), or anti-IgM antibody (IL-4:IgM). Then, microvesicles (MVs) and exosomes were collected by differential centrifugation (Fig. S1A) and analyzed by using antibodies against autophagosome markers (LC3-II and p62) and EV markers (ALIX, CD63, and CD81) and an endoplasmic reticulum marker (calnexin (CNX)). IL-4:CD40 co-stimulation induced higher secretion of p62^+^:LC3-II^+^ MVs and exosomes than IL-4:LPS and IL-4:IgM (Fig. 1A). Among Th1 and Th2 cytokines, IL-4 most potently induced ALV secretion (Fig. 1B), suggesting that ALVs could be secreted during B cell and Th2 CD4^+^ T cell interaction. To test this, B cells isolated from GFP-LC3 (GFP-LC3^+^) transgenic mice were co-cultured with Th cells differentiated from naïve CD4^+^ T cells of GFP-LC3-negative WT mice. Consistently, B cells co-cultured with Th2 CD4^+^ T cells induced greater ALV secretion than Th1 CD4^+^ T cells (Fig. 1C). ALV secretion was also found in GFP-LC3-expressing murine B-lymphoma A20 (GFP-LC3–A20) cells and IL-4:CD40 co-stimulation induced greater ALV secretion than IL-4:LPS co-stimulation (Fig. 1D). Furthermore, transmission electron microscopy analysis confirmed that GFP-LC3 protein, which was labeled by anti-GFP immunogold, was abundant in MVs and exosome fractions derived from IL-4:CD40-activated GFP-LC3^+^ B cells (Fig. 1E). Together, these results indicate that IL-4:CD40 co-stimulation induced p62^+^:LC3-II^+^ ALV secretion by B cells.

### ALVs secretion occurs through plasma membrane engulfing and budding

**Figure 2.**
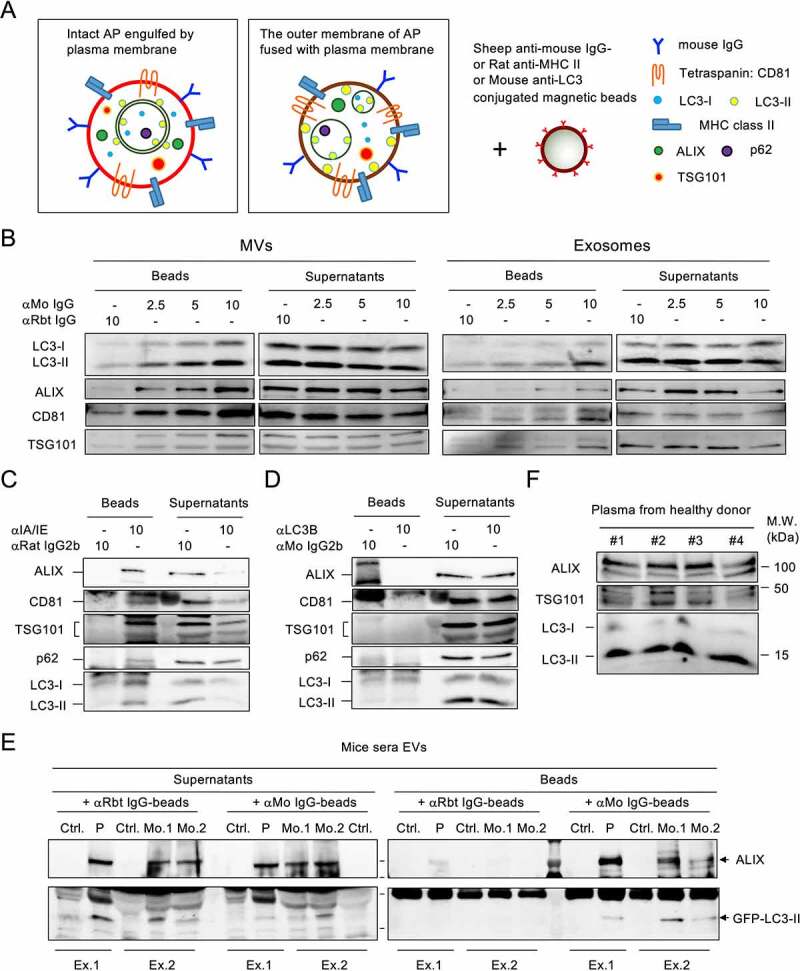
Fused-type ALVs circulate in the bloodstream **(A)** Proposed types of ALVs during plasma membrane shedding or budding. Left, autophagosome is engulfed by the plasma membrane. Right, the outer membrane of autophagosome is fused with plasma membrane. **(B)** Exosomes and MVs from IL-4:CD40-stimulated B cells were incubated with anti-mouse (αMo) IgG-conjugated beads or control beads. Washed beads bound to EVs and supernatants obtained after capturing were analyzed by immunoblotting using Abs against LC3, ALIX, CD81, and TSG101. Anti-rabbit (αRbt) IgG-conjugated beads were used as capturing control beads. **(C, D)** MVs from IL-4:CD40-stimulated B cells were incubated with anti-MHC class II-conjugated beads (C) or anti-LC3-conjugated beads (D). Washed beads bound to EVs and supernatants obtained after capturing were analyzed by immunoblotting using Abs against p62, LC3, ALIX, CD81, and TSG101. Isotype Abs-conjugated beads were used as capturing control beads. **(E)** EVs from sera of mice bearing GFP-LC3–A20 tumors were captured by αMo IgG beads, and both beads and supernatants were analyzed by immunoblotting using Abs against LC3 and ALIX. Ctrl.: control mice; P: pooled sera from three tumor-bearing mice; Mo.: individual tumor-bearing mouse; Ex: individual experiment. **(F)** EVs were purified from equal plasma volumes of four healthy human donors and analyzed by immunoblotting using Abs against ALIX, TSG101, and LC3.

It has been reported that autophagic cargo is released during the secretion of EVs and particles, particularly in small EVs [16,17]. Since p62 and LC3-II are abundant in MVs derived from IL-4:CD40-activated B cells, and the diameter of autophagosomes is thought to vary from a few hundred nanometers to over a micrometer [18], intact autophagosomes might be directly secreted by cells via plasma membrane engulfing or via fusion of the outer membrane of the autophagosome with the plasma membrane (Fig. 2A). Given the fact that IL-4:CD40 co-stimulation induced immunoglobulin G (IgG) class switching and membrane translocation [19], both types of ALVs may be immunoprecipitated by anti-mouse IgG-conjugated beads (αMo IgG beads) (Fig. 2A). As shown in Fig. 2B, LC3-II was detected in both MVs and exosomes captured by αMo IgG beads, but not by control αRabbit IgG beads (αRbt IgG beads). It has been reported that major histocompatibility complex (MHC) class II was localized on the surface of B cell-derived exosomes [20]. Since MHC class II was abundant in MV-type ALVs (Fig. 1B), and LC3-II may be localized on the surface of ALVs after membrane fusion, anti-mouse MHC class II-conjugated beads (αMHC-II beads) and anti-LC3-II-conjugated beads (αLC3-II beads) were used to characterize the identity of ALVs. As shown in Fig. 2C, LC3-II, and to a lesser extent p62, was detected in MVs captured by αMHC-II beads, suggesting that LC3-II^+^ALVs were separated from p62-containing cargos. However, neither the vesicle markers, ALIX or LC3-II nor p62 could be found in the MVs captured by αLC3-II beads, suggesting that LC3-II was localized inside the ALVs (Fig. 2D). Taken together, the majority of LC3-II^+^ MV secretion occurs through cell surface membrane engulfing or fusion and budding. The precipitated p62-containing cargos, particularly as membrane-free protein aggregates [21], during MV preparation may be secreted via an unknown pathway.

### Circulating ALVs in the bloodstream

We next took advantage of this isolation method to investigate whether ALVs could be secreted *in vivo* by activated B cells such as B-lymphoma cells. BALB/c mice were subcutaneously inoculated with surface IgG^+^GFP-LC3–A20 cells and serum EVs were purified from tumor-bearing mice. Similar to previous reports in which the number of serum EVs was increased in human patients with various tumors [22,23], the number of sera ALIX^+^ EVs was increased in tumor-bearing mice compared to control mice (Fig. 2E), see supernatant panel and control αRbt IgG bead treatment. Notably, GFP-LC3-II^+^ EVs were precipitated by αMo IgG beads but not αRbt IgG beads (Fig. 2E), indicating that these EVs were derived from GFP-LC3–A20 tumors. Additionally, a small number of endogenous serum GFP-LC3-II^+^ EVs was found in control mice, as previously reported [7], suggesting that GFP-LC3-II^+^ EVs can be secreted under normal physiological conditions. In agreement with these findings, LC3-II^+^ EVs were found in plasma from healthy human donors (Fig. 2F). These results indicate that ALVs circulate in the bloodstream under both normal physiological conditions and during tumor formation.

### Decrease of fusion activity between autophagosomes and lysosomes contributes to ALV secretion

**Figure 3.**
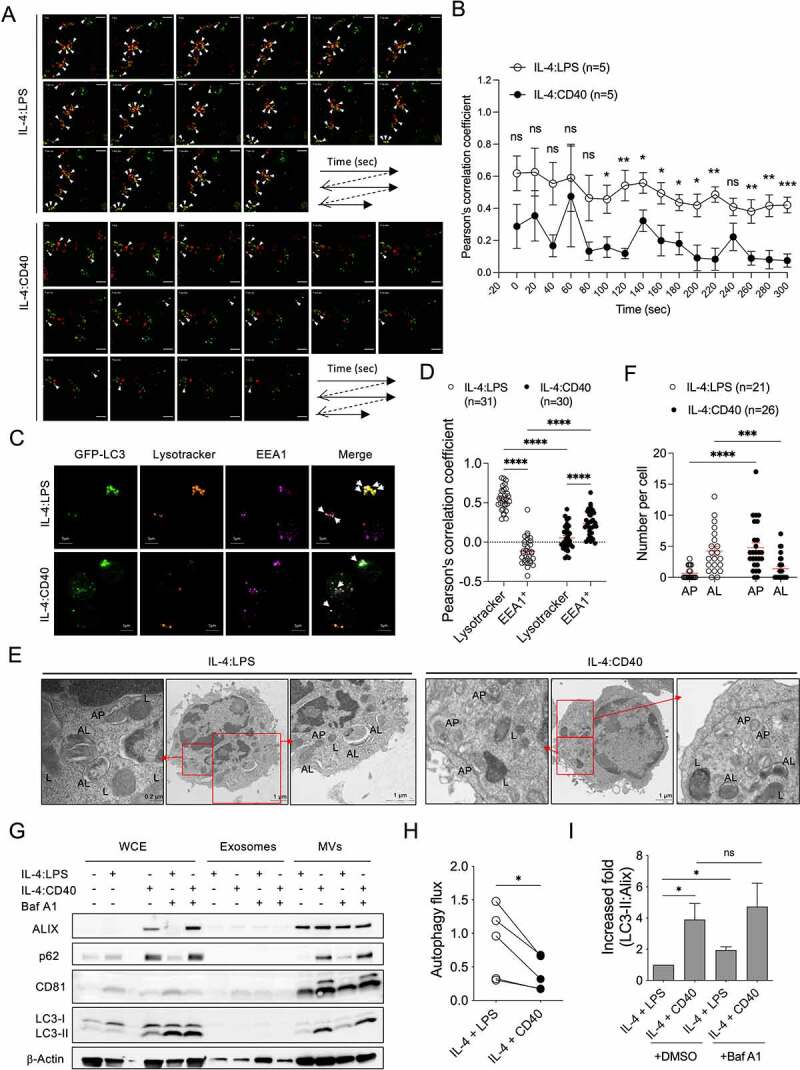
Fusion activity between autophagosome and lysosome contributes to ALV secretion **(A)** Representative confocal images of GFP-LC3^+^ dots (green) and lysotracker-labeled lysosomes (red) at different time points in GFP-LC3^+^ primary B cells stimulated with IL-4:LPS or IL-4:CD40 for 3 days. The white arrowheads indicate colocalization of GFP-LC3^+^ (green) and lysotracker^+^ (red) dots. Scale bar, 5 µm. Similar results were observed from two independent experiments. **(B)** The colocalization analysis in (A). Pearson’s correlation coefficient was used for quantifying the colocalization between GFP-LC3 and lysotracker at each time points. n = 5 GFP-LC3^+^lysotracker^+^ cells. **(C)** Representative fluorescence images from GFP-LC3^+^ primary B cells stained with lysotracker and anti-EEA1 Ab after 4 days of IL-4:LPS or IL-4:CD40 co-stimulation. Similar results were observed from two independent experiments. The white arrows indicate colocalization of GFP-LC3^+^ and lysotracker^+^ dots or EEA1^+^ dots in IL-4:LPS-stimulated or IL-4:CD40-stimulated cells, respectively. Scale bars, 5 μm. **(D)** The colocalization analysis in (C). Pearson’s correlation coefficient was used for quantifying the colocalization. **(E)** Representative TEM images of primary B cells stimulated with IL-4:LPS or IL-4:CD40 for 4 days. 7-AAD^−^ B cells were sorted using a cell sorter and analyzed by TEM. AL: autophagosome; L: lysosome; AP: autophagosome. Scale bars, 1 μm. Similar results were observed from two independent experiments. **(F)** Quantification of AP and AL numbers in (E). **(G)** Primary B cells were stimulated with indicated stimuli for 4 days. Then, cells were harvested and equal cell numbers were treated with 200 nM Baf A1 for another 4 to 5 h prior to the isolation of exosomes and MVs. WCE, isolated exosomes, and MVs were analyzed by immunoblotting using Abs against ALIX, CD81, SQSTM1/p62, LC3, and β-Actin. **(H)** Quantification of autophagic flux in **(G)**. Autophagic flux was calculated by subtracting the densitometric value of LC3-II normalized with loading control in DMSO-treated samples from that of Baf A1-treated samples. Data were pooled from five independent experiments. **(I)** Fold increases in the densitometric values of LC3-II normalized with ALIX in the MV fractions of **(G)**. Data were pooled from three independent experiments. Data are presented as mean ± SEM **(B, D, F, and I)** or before-after with symbols and lines **(H)**. Two-tailed unpaired t-tests **(B and I)**. Two-way ANOVA plus Šídák’s multiple comparisons test **(D)**. Mann-Whitney test **(F)**. Two-tailed paired t-tests **(H)**. ns: not significant, *p < 0.05, **p < 0.01, ***p < 0.001.

To investigate how IL-4:CD40 signals induce LC3-II^+^ ALV secretion in B cells, activated GFP-LC3^+^ B cells were labeled with lysotracker, and the fusion of autophagosomes (GFP-LC3^+^ dot) and lysosomes (lysotracker^+^ dot) was observed by high-resolution live-cell confocal microscopy. Live imaging analysis showed that autophagosome-lysosome fusion occurred less frequently in IL-4:CD40-stimulated than IL-4:LPS-stimulated B cells (Fig. 3A and 3B). While most autophagosomes were co-localized with lysotracker^+^ lysosomes in IL-4:LPS-stimulated B cells, autophagosomes were partially co-localized with EEA1^+^ early/late endosomes in IL-4:CD40-stimulated B cells (Fig. 3C and 3D). The increase of autophagosome-lysosome fusion in IL-4:LPS-stimulated B cells may not contribute to the increase of lysosomal biogenesis since both co-stimulations induced normal expression of LAMP2 [24], a lysosomal membrane protein (Fig. S1B). Additionally, EEA1 expression was higher in the IL-4:CD40-stimulated than IL-4:LPS-stimulated B cells (Fig. S1B), suggesting that the accumulation of early/late endosomes may increase their fusion with autophagosomes. TEM analysis also revealed that the number of autolysosomes was lower in IL-4:CD40-stimulated than IL-4:LPS-stimulated B cells (Fig. 3E and 3F). IL-4:LPS co-stimulation consistently enhanced autophagic flux to a greater extent than IL-4:CD40 co-stimulation (Fig. 3G and 3H), suggesting that high autophagic flux activity decreased the secretion of ALVs. As expected, suppression of autophagosome-lysosome fusion by bafilomycin A1 (Baf A1) enhanced LC3-II^+^ MV secretion in IL-4:LPS-stimulated B cells, but not in IL-4:CD40-stimulated B cells (Fig. 3G and 3I). The exosome fraction did not exhibit ALIX, p62, CD81, or LC3-II signals, which may have been due to the short duration of Baf A1 treatment (4-5 hours, Fig. 3G). These results suggest that a low frequency of IL-4:CD40-induced autophagosome-lysosome fusion may contribute to ALV secretion through the endosomal or MV shedding secretion pathways.

### RAB27a mediates ALV secretion

**Figure 4.**
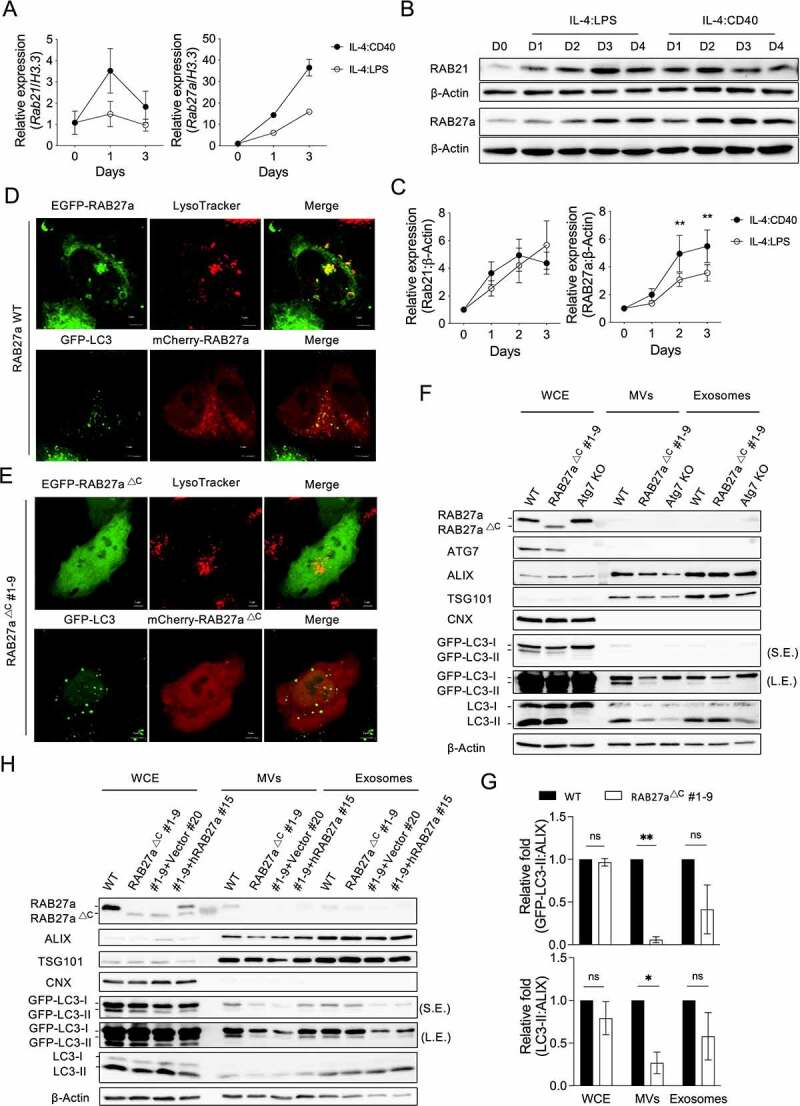
RAB27a mediates ALV secretion **(A)** RT-qPCR analysis of *Rab21* and *Rab27a* from primary B cells stimulated with IL-4:LPS or IL-4:CD40 for the indicated times. The quantitative data are presented as mean ± SD from three technical repeats. Similar results were observed from four independent experiments. **(B)** Representative immunoblotting of RAB21 and RAB27a proteins in primary B cells stimulated with IL-4:LPS and IL-4:CD40 for the indicated times. The numbers below indicate the protein levels normalized with loading control β-Actin. **(C)** Densitometric quantification of RAB21 and RAB27a expression in **(B)**. Data were pooled from six independent experiments. **(D)** EGFP-RAB27a or mCherry-RAB27a plasmid was transfected into either WT or GFP-LC3^+^ Hela cells for 24 h. Lysosomes were labeled by lysotracker 30 min prior to live-cell observation using a confocal microscope. Scale bar, 5 μm. **(E)** EGFP- or mCherry-RAB27a^∆C^ #1-9 plasmids were transfected into either WT or GFP-LC3^+^ Hela cells for 24 h. Lysosomes were labeled by lysotracker 30 min prior to live-cell observation using a confocal microscope. **(F)** Equal numbers of WT, RAB27a^∆C^, or ATG7 KO GFP-LC3–A20 cells were cultured for 72 h prior to isolation of the exosomes and MVs from supernatants. WCE, isolated exosomes, and MVs were analyzed by immunoblotting using Abs against ATG7, ALIX, TSG101, CNX, LC3, and β-Actin. S.E. and L.E. indicate short and long exposure, respectively. **(G)** Fold changes of the densitometric values of GFP-LC3 and LC3-II normalized with ALIX in the WCE, exosomes, and MV panels of **(F)**. Data were pooled from three independent experiments. (**H)** RAB27a^∆C^ #1-9 A20 cells were reconstituted with human WT RAB27A. Equal numbers of the indicated cells were cultured for 72 h prior to isolation of the exosomes and MVs from supernatants. WCE, isolated exosomes, and MVs were analyzed by immunoblotting using Abs against ALIX, TSG101, CNX, LC3, and β-Actin, respectively. Data are presented as mean ± SEM **(C, G)**. Both factors-matched two-way repeated measurement ANOVA with Šídák’s multiple comparisons test **(C)**. Two-tailed paired t-tests **(G)**. ns: not significant, *p < 0.05, **p < 0.01.

We next used real-time quantitative reverse-transcription PCR (RT-qPCR) to investigate the gene expression involved in autophagosome-lysosome fusion and vesicle transportation in activated B cells (Fig. S2A). Compared to IL-4:LPS signals, IL-4:CD40 signals induced lower expression of genes involved in autophagosome-lysosome fusion or vesicle transportation, such as *stx5a, stx7, stx8, stx18, rab7, vamp7, vamp8, sec22b, rab3a, rab4b, rab5c, rab8a, rab11a, rab15, rab23*, and *rab26*. (Fig. S2B and S2C). However, *Rab21* and *Rab27a* expression was higher in IL-4:CD40-stimulated than IL-4:LPS-stimulated B cells (Fig. 4A). Immunoblotting confirmed that IL-4:CD40 co-stimulation induced the expression of RAB27a but not RAB21 (Fig. 4B and 4C). Additionally, RAB27a not only co-localized with lysotracker^+^ lysosomes as previous reported, but also partially co-localized with GFP-LC3^+^ autophagosomes (Fig. 4D), suggesting that RAB27a may mediate autophagosome transportation during IL-4:CD40 co-stimulation. To elucidate the function of RAB27a in ALV secretion, we used the CRISPR-Cas9 gene editing system and the GFP-LC3–A20 cell line to generate a RAB27a mutant lacking the CXC motif (RAB27a^ΔC^) (Fig. S3A). An *Atg7* knockout (KO) GFP-LC3–A20 cell line was also generated as a control. Overexpression of RAB27a^ΔC^ resulted in even distribution inside Hela cells (Fig. 4E), confirming that the CXC motif is required for RAB27a targeting to specific vesicles [25]. Interestingly, the GFP-LC3-II and endogenous LC3-II proteins, but not the ALIX protein, were decreased in MVs but not exosomes derived from the RAB27a^ΔC^ cells (Fig. 4F and 4G), suggesting that RAB27a, via the MV shedding pathway, is the predominant mediator of autophagosome secretion in B cells. In agreement with this, reconstitution of human wild-type (WT) RAB27A reversed the decrease of ALV secretion in MVs derived from RAB27a^ΔC^ cells (Fig. 4H). Taken together, these results suggest that IL-4:CD40-induced RAB27a expression mediates ALV secretion in B cells.

## Discussion

Previous reports showed that suppression of autophagosome-lysosome fusion enhanced ALV secretion. Whether ALVs can be secreted during cell activation remained unclear. Here, we demonstrated that IL-4:CD40 co-stimulation activated autophagy but reduced the autophagosome-lysosome fusion, and thus promoted the secretion of LC3-II^+^ ALVs in B cells. During pathogen infection or vaccination, follicular Th (TfH) cells secrete cytokines, present antigens, and provide co-stimulatory signals to facilitate germinal center B-cell proliferation and differentiation [11,12]. Among TfH cytokines, IL-4 was reported to effectively induce autophagy in B cells [15]. However, IL-4 autophagy induction was insufficient for maintaining long-term B-cell survival. Co-stimulatory signals such as those mediated by TLRs, B-cell receptors, and CD40 receptors, are required to provide survival signals through different NF-κB complexes [26-28], including NF-κB1/c-Rel heterodimers, NF-κB1/RelA, NF-κB1 homodimers, c-Rel/RelA, NF-κB2/RelB, and NF-κB2 homodimers. LPS, a ligand for TLR4, rapidly induces classical NF-κB1 signaling that recruits NF-κB1/c-Rel, NF-κB1/RelA, and NF-κB1 homodimers from the cytoplasm to the nucleus [26,28]. CD40 signals not only activate the same NF-κB complexes as LPS signals [27,28], but also induce the expression of both RelB and c-Rel complexes to activate the alternative NF-κB2 pathway [26,28]. B-cell receptor signals activate both the NF-κB1 and the nuclear factor of activated T cells-dependent gene expression pathways [27,28]. Although IL-4:CD40, IL-4:LPS, and IL-4:IgM co-stimulation all induced autophagy, neither IL-4:LPS nor IL-4:IgM co-stimulation was sufficient for the secretion of ALVs, suggesting that the NF-κB2 signaling pathway is required for ALV secretion. Indeed, co-stimulation with IL-4:CD40 induced lower autophagic flux than with IL-4:LPS, suggesting that NF-κB2 signaling counteracts NF-κB1 signaling in the regulation of autophagic flux. Additionally, co-stimulation with IL-4:CD40 induced higher RAB27a expression than with IL-4:LPS. Thus, the NF-κB2 signaling pathway may regulate RAB27a expression and autophagic turnover, which are two key factors that promote ALV secretion.

It has been reported that RAB27a regulates intracellular vesicle transportation and secretory lysosomes [29-31]. Solvik et al. also showed that RAB27a mediates the exocytosis of autophagosome cargo receptors during lysosome inhibition [7]. Our findings identify a new role for RAB27a, specifically the regulation of ALV secretion during B-cell activation (Fig. S3B). Co-stimulation with IL-4:CD40 resulted in greater RAB27a expression than with IL-4:LPS in B cells. The presence of RAB27a on the autophagosome membrane may mediate the transportation of autophagosomes or amphisomes toward the plasma membrane. Although MVs and exosomes share similar mechanisms in biogenesis, the secretion of MVs depends on the shedding or budding of enclosed plasma membranes with recruited cargos, whereas exosomes are secreted by the fusion of multivesicular bodies with recruited cargos [32]. Since the loss of membrane-bound RAB27a impairs MV-type ALV secretion without affecting the secretion of other vesicles, RAB27a may facilitate ALV release by directly guiding autophagosomes or amphisomes as they dock with the plasma membrane, followed by exosome secretion or membrane shedding. However, further investigation is needed to identify the detailed mechanism.

Accumulating evidence has shown that EV play an important role in the regulation of functions of neighboring immune cells [33]. B cell-secreted exosomes containing MHC class II-peptide complexes could induce antigen-specific T cell responses [20]. Increased secretion of MHC II^+^LC3-II^+^ EVs during B-T cell interaction may also influence T and/or B cell activity. Although Th2 immune response is necessary for activating B cells during helminth infection [34], its initiation against harmless allergens will lead to atopic dermatitis or food allergy [35,36]. B cell secreted LC3-II^+^ EVs might either drive protective immune response or promote disease development. Additionally, it has been reported that LC3-II^+^ EVs carried biological messages, such as RNA-binding proteins, small nucleolar RNAs and microRNAs (miR) [5]. B cell-derived LC3-II^+^ EVs may contain specific small RNAs to regulate the function of neighboring cells, such as B, T or antigen-presenting cells [33]. For example, miR-363 enriched from IL-4:CD40-activated B lymphoma cell-derived EVs enhanced migration, proliferation and immunological synapse signaling of autologous CD4^+^ T cells [37]. Exploring the biological information carried by B cell-derived ALVs may help us understand their positive or negative roles in the regulation of neighboring cells.

Several studies have shown that autophagy is required for the digestion of tumor antigens, and that it results in effective immune surveillance that facilitates tumor survival [38,39]. Vaccination with autophagosomes isolated from tumor cells induced cross-protective immune responses against tumors [40,41]. Our results reveal that activated B cells, such as B-lymphoma cells, secrete ALVs into the bloodstream. Since ALVs are found in healthy human plasma, secretion of ALVs may be increased in patients with B-cell lymphoma. Thus, isolation of ALVs from such patients using specific B-cell markers may help to identify novel neo-antigens for vaccine design.

In this study, we demonstrated that external IL-4:CD40 co-stimulation enhanced ALV secretion by B cells through a RAB27a-dependent pathway. While it is known that exosome secretion is required for early B-cell development in bone marrow [42,43], our findings may reveal a new role for secretory autophagy in the regulation of intercellular communication during B-cell activation in the context of vaccination, autoreactivity, tumorigenesis, and pathogen infection.

## Material and methods

### Mice, tumor inoculation and serum collection

C57BL/6J and BALB/c mice, 8-12 weeks of age, were purchased from CLEA, Japan. GFP-LC3 transgenic mice [44] were kindly provided the RIKEN BRC through the National Bio-Resource Project of the MEXT, Japan. Mice were housed in microisolator cages with autoclaved food and bedding to minimize exposure to microbial pathogens. All animal care procedures were performed in accordance with the Standards Relating to the Care and Management of Laboratory Animals and Relief of Pain formulated by the Ministry of the Environment, Japan. The protocols of animal experiments were approved by the Animal Experimentation Committee of the Research Institute for Microbial Disease, Osaka University.

For serum collection from tumor-bearing mice, 3×10^6^ GFP-LC3–A20 cells in 100 µl of Dulbecco’s Phosphate Buffered Saline (PBS, Nacalai, 14249-95) were inoculated subcutaneously into BALB/c mice. Once a tumor formed (< 1000 mm^3^), blood was collected from the tail into a 1.5-mL tube and allowed to clot for 30 min at room temperature (RT). Sera were centrifuged at 2000 × g for 20 min to eliminate cell debris and fragments, and at 8000 × g for 30 min to eliminate large debris, then stored at -80 °C.

### Human plasma

Blood samples were collected from healthy donors at Osaka University Hospital (Osaka, Japan), according to the protocol approved by Osaka University Research Ethics Committee.

### Primary B-cell isolation and stimulation

Spleens from C57BL/6J mice were placed in HBSS(+) (Nacalai, 17459-55) containing 2% fetal bovine serum (FBS, Gibco, 10270106) and 1% penicillin-streptomycin (Nacalai, 26252-94), cut into small pieces, and ground with the frosted surface of microscope glass slides (MATSUNAMI, S2215) until the pieces were transparent. The spleen cell suspensions were filtered through a 100-µm strainer (Greiner Bio-One, 542000) into a new tube, then centrifuged at 300 x g for 5 min at 4 °C. After discarding the supernatants, cells were suspended in R10 (RPMI-1640 [Wako; 189-02025] supplemented with 10% FBS, 1% penicillin-streptomycin, non-essential amino acids [NEAA, Nacalai, 06344-56], and 2-mercaptoethanol [Gibco, 21985023]). Naïve primary B cells were isolated by negative selection using an isolation kit (Miltenyi Biotec, 130-090-862). B cells were stimulated with the indicated combinations using anti-mouse CD40 antibody (1 µg/ml, Biolegend, 102902), F(ab’)₂ fragment goat anti-mouse IgM antibody, µ-chain-specific (1 µg/ml, Jackson ImmunoResearch Laboratories, 115-006-020), lipopolysaccharides (1 µg/ml, Sigma-Aldrich, L2640), IL-1β (10 ng/ml, R&D Systems, 485-ML), IL-4 (10 ng/ml, Peprotech, 214-14), IL-5 (10 ng/ml, R&D Systems, 447-ML), IL-6 (10 ng/ml, R&D Systems, 406-ML), IL-12 (10 ng/ml, R&D Systems, 419-ML), IL-21 (10 ng/ml, R&D Systems, 594-ML), and IFN-γ (10 ng/ml, R&D Systems, 485-ML). For the suppression of autophagosome-lysosome fusion, Baf A1 (200 nM, BioViotica, BVT-0252-M001) was added to stimulated cells for another 4-5 h.

### Cell coculture assay

Naïve T cells were isolated by negative selection using an isolation kit (Miltenyi Biotec, 130-104-453). Cells were labeled with antibodies against CD44 (Biolegend, 103007), CD62L (Biolegend, 104412), and CD4 (Biolegend, 100405). CD4^+^CD62L^+^CD44^−^ population was sorted by using FACSAria™ II (BD bioscience). The culture medium was supplemented with Dynabeads™ Mouse T-Activator CD3/CD28 (Gibco, 11-452-D), cytokines and blocking antibodies accordingly. Th1: IL-12 (10 ng/ml, R&D Systems, 419-ML) and anti-IL-4 antibody (10 µg/ml, Biolegend, 504122). Th2: IL-4 (10 ng/ml, Peprotech, 214-14) and anti-IL-12 antibody (10 µg/ml, Biolegend, 511802). After 4 days incubation, activation beads were removed and differentiated Th cells were seeded in the culture dishes coated with anti-CD3ε antibody (10 µg/ml, Biolegend, 100302) and co-cultured with GFP-LC3^+^ B cells at a ratio of 1:1 in the culture media supplemented with anti-CD40 antibody (1 µg/ml, Biolegend, 102902). After 2 days co-culture, the culture medium was collected and used for EV preparation described blow.

### Cell lines

GFP-LC3–A20 cells were cultured in R10 medium. WT and GFP-LC3–expressing Hela and Platinum-E cells were kindly provided by Dr. Takeshi Noda (Osaka University, Japan) and maintained in D10 (D-MEM [Wako, 044-29765] supplemented with 10% FBS and 1% penicillin-streptomycin). Platinum-E cells were maintained with D10 supplemented with Blastidine (10 μg/ml) and puromycin (1 μg/ml). All cells were incubated at 37 °C in a humidified atmosphere of 5% CO and 95% air.

### Plasmid construction

To generate gRNA expression vector, the forward and reverse oligonucleotide of gRNA targeting *Rab27a* (forward: 5’-CACCGAGGGAGTGGTACGGTCCAA-3’, reverse: 5’-AAACTTGGACCGTACCACTCCCTC-3’) and *Atg7* (5’-CACCGAACTCCAACGTCAAGCGGGT-3’, reverse: 5’-AAACACCCGCTTGACGTTGGAGTTC-3’) were 1:1 mixed in TE buffer and incubate at 94°C for 5 min, then placed at 60°C for 5min. The annealed oligos were cloned into px459 vectors [45] by using ligation kit (Toyobo, LGK-201).

To generate plasmid containing of murine and human RAB27A, following primers were used: Forward primer for murine WT and mutant *RAB27a* is 5’-CAGATCTTCGGATGGAGATTACGATTAC-3’ and reverse primer for murine WT *RAB27a* is 5’-CGTCGACTCAACAGCCACACAACCCC-3’. Reverse primer for murine mutant RAB27a is 5’-CGTCGACTCACTTAGCTGATCCGCAG-3’. Forward and reverse primer for hRAB27A are 5’-CAGATCTCATTATGTCTGATGGAGATTATG-3’ and 5’-CGTCGACTCAACAGCCACATGCCCCTT-3’, respectively.

cDNA from A20 or Hela was used as template and targeted gene cDNA was amplified by using KOD Neo FX (Toyobo, KFX-201). The amplified products were purified by using PureLink™ Quick Gel Extraction Kit (Invitrogen, K210012). The PCR products were digested with restriction enzyme *Bgl*II (Takara, 1021A) and *Sal*I (Takara, 1080A) according to manufacturer’s condition and cloned into pEGFP-C1, pmCherry-C1 and MSCV-IRES-Thy1.1, respectively. All plasmids were isolated using PureLink™ HiPure Plasmid Midiprep Kit (Invitrogen, K210004) according to manufacturer’s instruction.

### Generation and maintenance of mutant cell lines

For generating *Atg7* KO and *Rab27a* mutant cells, px459 plasmids carrying gRNA were electroporated into GFP-LC3^+^ A20 cells using the Neon transfection system (Thermo Fisher Scientific) with the following condition: 7.6×10^6^/ml, 100-µl tip, 1400 V, 30 ms, and 1 pulse, then cultured in R10 for 24 h. The cells were treated with 1 µg/ml puromycin for another 24 h prior to single-cell sorting using a FACSAria III (BD Bioscience). The dead cells were excluded by staining cells with 7-AAD (Biolegend, 420404). ATG7 and RAB27a protein expression was confirmed by immunoblotting.

To generate hRAB27A-restored cell lines, retroviruses carrying hRAB27A were produced using Platinum-E cells transfected with retroviral vector MSCV-IRES-Thy1.1 containing hRAB27A or empty vector in the presence of Lipofectamine3000 reagent (Thermo Fisher Scientific, L3000015). Two days later, supernatants were collected and filtered with a 0.22-μm filter (Merck, SLGVR33RS). RAB27a ∆C #1-9 was transduced by retroviruses using the spin-infection method (3,000 x g for 90 min at 24 °C). One day later, culture media were replaced with R10 for another day and cells were stained with anti-Thy1.1 antibody (Biolegend, 202522, 1:250) and 7-AAD and subjected to single-cell sorting. hRAB27A protein expression was confirmed by immunoblotting.

### EV preparation

Cell culture supernatants were centrifuged at 200 x g and 2000 x g to remove cells and cell debris, respectively. The supernatants were collected and centrifuged at 15,000 x g for 40 min at 4 °C to pellet MVs. Clear supernatants were filtered through 0.22-µm filters and then ultracentrifuged (Beckman Coulter L-100XP Ultracentrifuge) at 120,000 x g for 80 min at 4 °C to pellet exosomes. MVs and exosomes were suspended either in PBS for immune-isolation and EM, or dissolved in cell lysis buffer (CLB, Cell Signaling Technology, 9803) with protease inhibitor cocktail (Halt™ Protease Inhibitor Cocktail [100X], ThermoFisher Scientific, 87786) for immunoblotting.

For EVs from mouse sera, 60 µl of serum was incubated with 12.6 µl of ExoQuick (System Biosciences, EXOQ5A-1) on ice for 30 min, then centrifuged at 1,500 x g for 30 min and 5 min to remove all fluids. The pellets were resuspended either in PBS for immune-isolation, or CLB with protease inhibitor for western blotting.

For EVs from human plasma, 2 ml of human plasma was incubated with human-derived thrombin (Nacalai, 33839-46) for 10 min to convert fibrinogen to fibrin. Samples were centrifuged briefly and clear supernatants were transferred into new tubes and filtered with 0.45-µm PVDF filters (Merck, SLGVJ13SL). The EVs from human plasma were obtained by ultracentrifugation and dissolved in CLB with protease inhibitor for immunoblotting.

### RNA isolation and RT-qPCR

RNA was isolated by using the RNeasy Micro Kit (Qiagen, 74004), and an equal amount of RNA was reversed transcribed into complementary DNA using the SuperScript™ VILO™ cDNA Synthesis Kit (Invitrogen, 11754). One RT-qPCR reaction mixture was prepared as a total volume of 10 µl containing qPCR Mix and ROX (Toyobo, QPS-201), distilled water, sample cDNA, and primers (see Supplemental table 1). The reaction was performed using a StepOne Plus system (Applied Biosystems). Relative expression of mRNA levels was normalized with the expression of an internal control, H3.3.

### Western blotting and antibodies

For whole-cell lysates, 1x10^6^ cells were lysed in 40 µl CLB supplemented with protease inhibitor cocktail and incubated on ice for 30 min. The lysates were centrifuged at 14,000 x g for 15 min at 4 °C to obtain supernatants. The protein concentration was determined using the DC Protein Assay Kit (Bio-Rad, 5000116). For EVs, isolated EVs were lysed in CLB and sonicated in order to enhance the protein extraction. All protein lysates were mixed with 5X sodium dodecyl sulfate (SDS) sample loading buffer and boiled for 10 min at 95 °C, then separated on SDS-PAGE gel (Bio-Rad, 4561096). Proteins were transferred to 0.2-μm nitrocellulose membranes (Bio-Rad, 1620097) and blocked with 5% bovine serum albumin (Sigma-Aldrich, A9647) in TBS-T buffer (20 mM Tris [Nacalai, 35434-21], 150 mM NaCl [Nacalai, 31320-05], 0.1% Tween20 [Bio-Rad, 161-0781]) for 1 h at RT. The membranes were probed with primary antibodies in blocking buffer overnight at 4 °C, then incubated with secondary antibodies (1:10,000 dilution) with 5% skim milk in 1X TBS-T for 1 h at RT. Protein bands were imaged using the Fujifilm LAS-4000 system with chemiluminescent substrate (Thermo Fisher Scientific, 34577, 34095 or A38555).

Primary antibodies used in this study were following: mouse anti-ALIX (BioLegend, 634501; 1:2,000); rat anti-mouse CD63 (BioLegend, 143902; 1:1,000); hamster anti-mouse/rat CD81 Antibody (BioLegend, 104901; 1:1,000), rabbit anti-CNX (Sigma-Aldrich, C4731; 1:2,000); HRP conjugate anti-β-Actin (Cell Signaling Technology, 3700S; 1:20,000); mouse anti-EEA1 (Cell Signaling Technology, 48453S; 1:1,000); rabbit anti-LAMP1 (Cell signaling Technology, 9091; 1:1,000); rabbit anti-LC3 (Cell Signaling Technology, 2775; 1:1,000); MHCII (Santa Cruz, sc59322; 1:1,000); rabbit anti-SQSTM1/p62 (Cell Signaling Technology, 23214; 1:1,000); rabbit anti-Rab21 (Invitrogen, PA5-88344; 1:1,000); rabbit anti-Rab27a (Cell Signaling Technology, 69295S; 1:1,000); rat anti-TSG101 (BioLegend, 934301; 1:1,000). Primary antibody: Peroxidase AffiniPure Goat Anti-Armenian Hamster IgG (H+L) (Jackson ImmunoResearch, 127-035-160; 1:10,000), Peroxidase AffiniPure Donkey Anti-Mouse IgG (H+L) (Jackson ImmunoResearch, 715-035-151; 1:10,000); Peroxidase AffiniPure Donkey Anti-Rat IgG (H+L) (Jackson ImmunoResearch, 715-035-150; 1:10,000); Peroxidase AffiniPure Donkey Anti-Rabbit IgG (H+L) (Jackson ImmunoResearch, 711-035-152; 1:10,000).

### Immuno-isolation of EVs

For immune-isolation using anti-IgG antibody coated beads, EVs were immunoprecipitated using Dynabeads™ M-280 Sheep Anti-Mouse IgG (Invitrogen, 11202D), and Dynabeads™ M-280 Sheep Anti-Rabbit IgG (Invitrogen, 11203D) was used for control. The indicated beads were washed twice with PBS and then incubated with 15 μl of EVs in PBS for 30 min at RT. For immuno-isolation using anti-MHCII and anti-LC3 antibody coated beads, anti-mouse I-A/I-E (BD bioscience, 556999), and anti-mouse LC3B (Cell Signaling Technology, 83506) were incubated with Dynabeads Protein G (Invitrogen, 10003D) for 30 min at RT. Purified rat IgG2b (Biolegend, 400621), and purified mouse IgG2b (Biolegend, 401202) were used for control. The indicated beads were washed twice with PBS and then incubated with equal amount of MVs or exosomes from IL4:CD40 co-stimulated primary B cell, for 30 min at RT. Supernatants were transferred to new tubes and beads were washed with PBS five times. Thirty microliters of CLB supplemented with proteinase inhibitors were added to bead-captured EVs. Bead-captured EVs and supernatants were mixed with 5X SDS sample buffer and boiled for 10 min at 95 °C for western blot analysis.

### Fluorescence imaging

For the live observation of GFP-LC3^+^ dots and lysososmes, GFP-LC3^+^ naïve B cells were stimulated with anti-mouse CD40 antibody or LPS combined with IL-4 for 3 days. The stimulated cells were harvested and cultured with Lysotracker™ Red DND-99 (Thermo Fisher Scientific, L7528; 200 nM) at 37 °C for 30 min prior to 3D time-lapse imaging using a FV3000 confocal microscope (Olympus Life Science). The projection images at each time points were analyzed by using cellSens software (Olympus Life Science) to obtain their Pearson’s coefficients.

To determining the locations of RAB27a, GFP-LC3^+^ dots, and lysosomes, WT or GFP-LC3^+^ Hela cells were seeded into a chambered coverslip (ibidi, µ-Slide 8 Well high, 80806) for 1 day prior to polyethylenimine transfection using GFP-WT RAB27a, mCherry-WT RAB27a, GFP-RAB27a mutant, and mCherry-RAB27a mutant plasmids. Twenty-four hours later, transfected cells were cultured with 200 nM Lysotracker™ Red DND-99 in D10 at 37 °C for 30 min prior to live-cell observation using an FV-3000 confocal microscope (Olympus Life Science).

To determine the locations of early/late endosome and lysosome in stimulated GFP-LC3^+^ B cells, cells were cultured with 200 nM Lysotracker™ Red DND-99 in R10 at 37 °C for 30 min prior to fix with 4% paraformaldehyde at 4 °C for 20 min. Fixed cells were permeabilized with digitonin (50 µg/ml, Wako, 044-02121) in PBS at RT for 30 min and blocked with 2 % gelatin and 2 % goat normal serum in PBS at RT for 30 min prior to stained with anti-EEA1 antibody (0.5 µg/ml, Cell Signaling Technology, 484535) at 4 °C overnight. Cells were then washed with PBS and stained with the fluorescent secondary antibody, F(ab’)2-Goat anti-Rabbit IgG (H+L)-A647 (Thermo Fisher Scientific, A-21246), for 60 min at RT. The cells were mounted with ProLong™ Gold Antifade Mountant (Thermo Fisher Scientific, P36930) and analyzed using an FV3000 (Olympus Life Science). Pearson’s coefficients were analyzed by using cellSens software (Olympus Life Science).

### Transmission electron microscopy

Live cells were sorted and fixed in 2% formaldehyde/2.5% glutaraldehyde/0.1 M phosphate buffer (pH 7.4) and washed gently with 4% sucrose/0.1 M phosphate buffer. Cells were post-fixed for 1 h with 0.1 M sodium-phosphate buffer (pH 7.4) supplemented with 1% osmium tetroxide and 0.5% potassium ferrocyanide, dehydrated using a graded ethanol series, and embedded in Epon812 (TAAB Co. Ltd.). Ultra-thin sections (80-nm thickness) were stained with saturated uranyl acetate and lead citrate solution. Electron micrographs were obtained with a JEM-1400plus transmission electron microscope (TEM, JEOL). A vacuole with an electron-dense membrane or multiple limiting membranes containing internal cytoplasm with the same morphology and electron density as the external cytoplasm indicated an autophagosome. A double-membrane or an inner-limiting-membrane vacuole containing electron-dense contents indicated an autolysosome. A single-outer-membrane vacuole containing electron-dense contents indicated a lysosome.

For immunogold labeling of EVs, EVs suspended in PBS were diluted 1:10 with PBS and fixed with 2% paraformaldehyde/PBS for 10 min. After washing with PBS, EVs were permeabilized with 0.005% Triton-100/PBS for 5 min. Then the EVs were blocked with 5% normal goat serum/PBS for 15 min and stained with 50X dilution of anti-GFP antibody (Abcam, ab290)/blocking buffer for 2 h. After washing with blocking buffer, EVs were incubated with 50X dilution of 10-nm gold-conjugated anti-IgG (H+L) antibody (BBI Solutions, EMRAG10)/blocking buffer for 50 min, followed by three washes with PBS and then 2% glutaformaldehyde fixation for 10 min. After fixation, samples were washed with PBS and H_2_O three times, then stained with 0.5% uranylacetate/H_2_O. After drying, images were obtained at a voltage of 80 kv using a JEM-1400plus transmission electron microscope (JEOL).

## Statistics

All statistical analyses were performed using GraphPad Prism 9 (GraphPad software). The tests used for statistical analyses and expressed values are described in the figure legends.

## Supplementary Material

Supplemental Material
