# Supplementary figures and images for: External stimulation induces the secretion of autophagosome-like vesicles by B cells
Source: Autophagy Rep. 2023 Feb 21;2(1):2179287. doi: 10.1080/27694127.2023.2179287 (PMC12039400; doi:10.1080/27694127.2023.2179287)

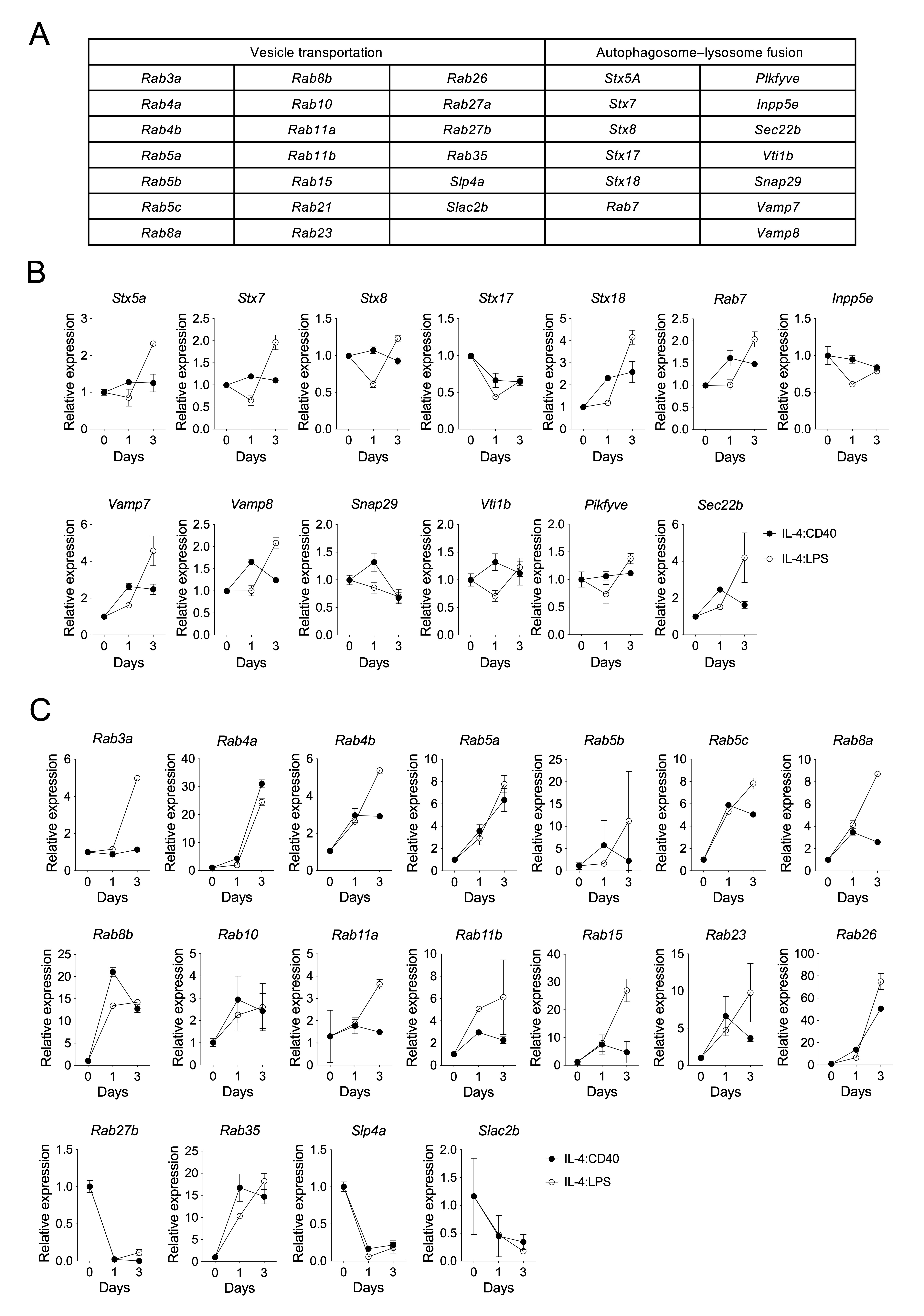

Supplement: Supplemental Material [file KAUO_A_2179287_SM1258.zip › Figure S2.jpg]

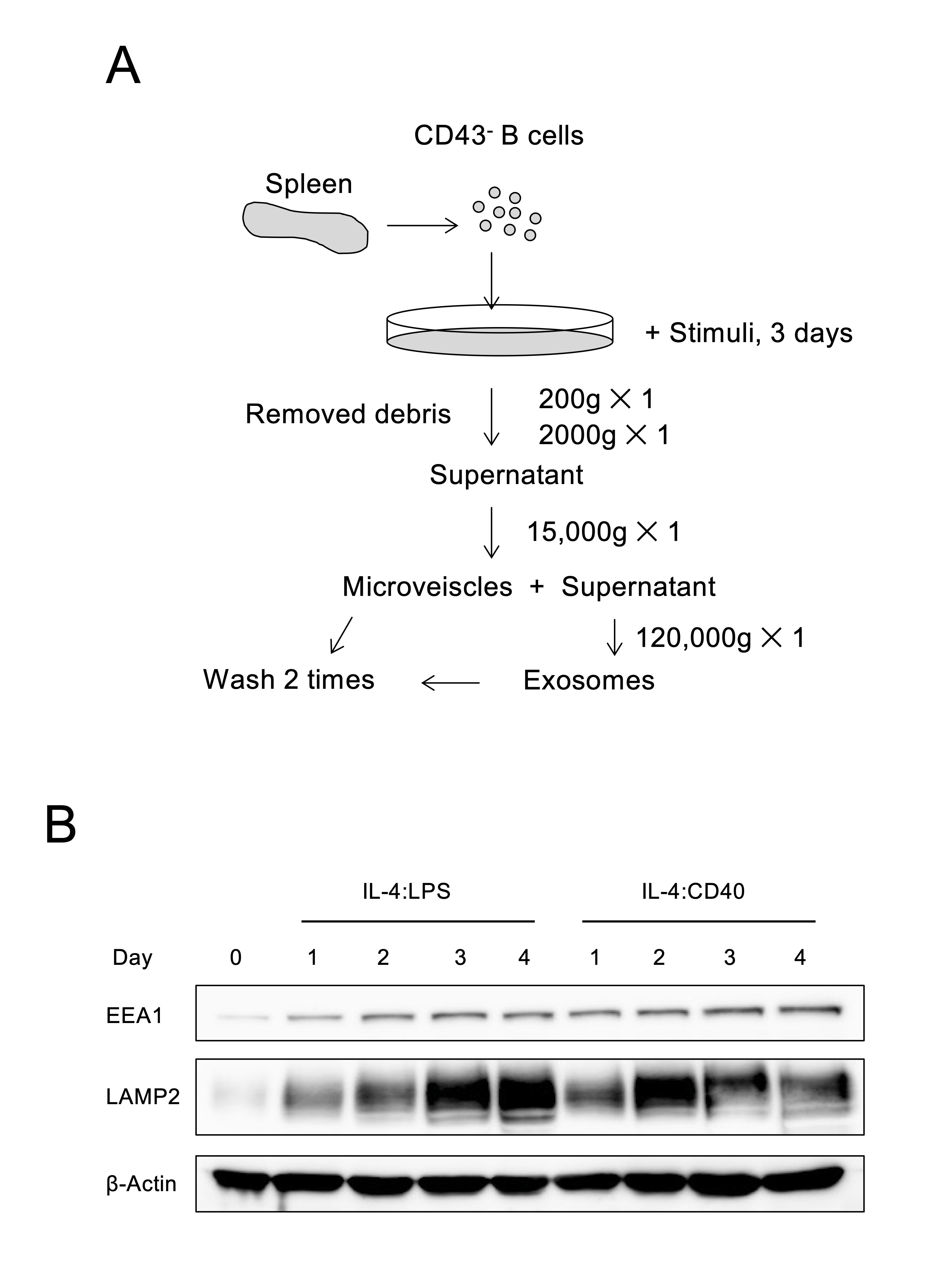

Supplement: Supplemental Material [file KAUO_A_2179287_SM1258.zip › Figure S1.jpg]

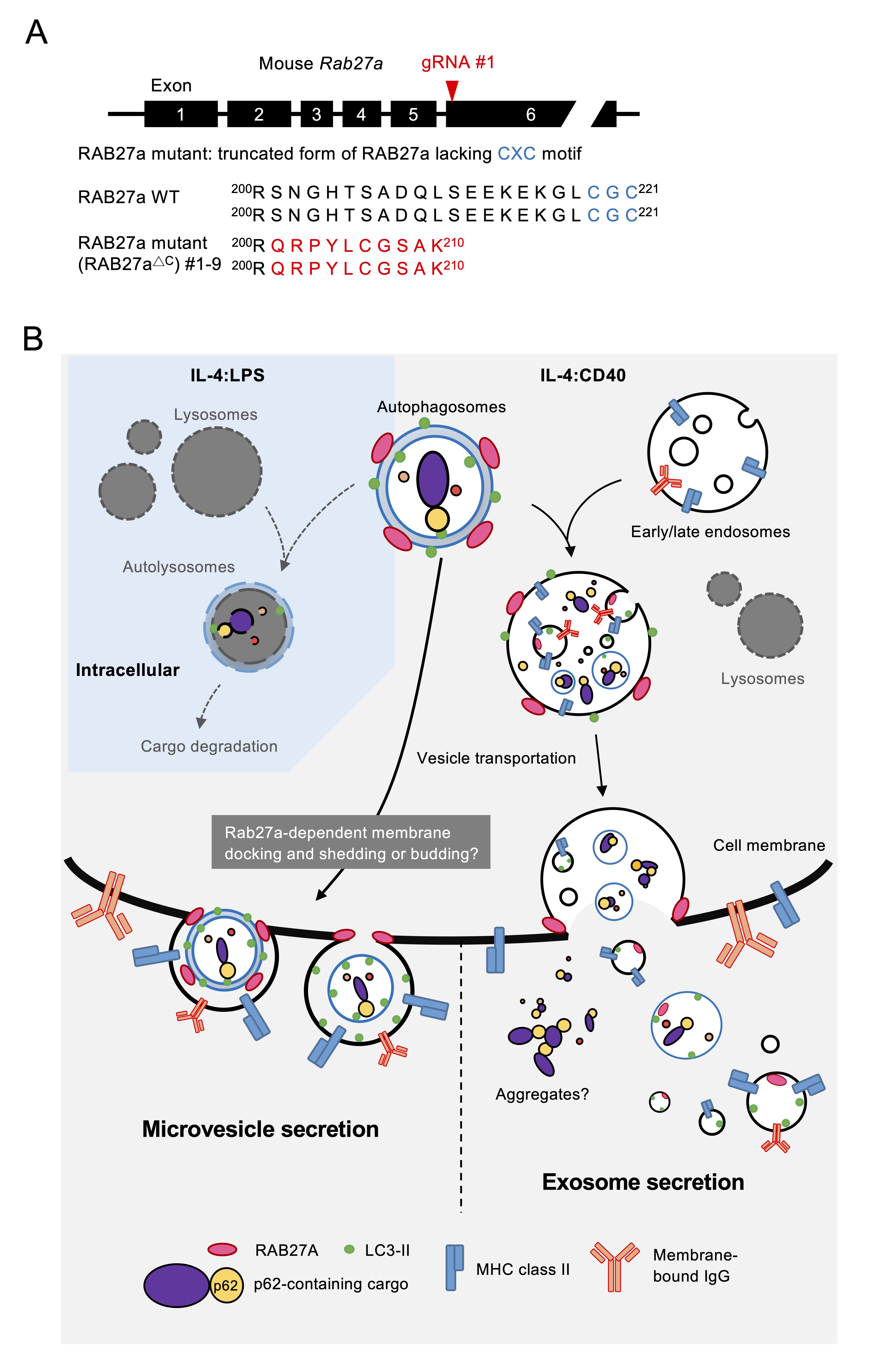

Supplement: Supplemental Material [file KAUO_A_2179287_SM1258.zip › Figure S3.jpg]
